# Patient decision aid based on multi-criteria decision analysis for disease-modifying drugs for multiple sclerosis: prototype development

**DOI:** 10.1186/s12911-021-01479-w

**Published:** 2021-04-09

**Authors:** I. E. H. Kremer, P. J. Jongen, S. M. A. A. Evers, E. L. J. Hoogervorst, W. I. M. Verhagen, M. Hiligsmann

**Affiliations:** 1 Department of Health Services Research, School CAPHRI Care and Public Health Research Institute, Maastricht University, Maastricht, The Netherlands; 2 MS4 Research Institute, Nijmegen, The Netherlands; 3 Department of Community and Occupational Medicine, University Medical Centre Groningen, Groningen, The Netherlands; 4 Public Mental Health, Trimbos Institute, Netherlands Institute of Mental Health and Addiction, Utrecht, The Netherlands; 5 Department of Neurology, St. Antonius Hospital, Utrecht, The Netherlands; 6 Department of Neurology, Canisius Wilhelmina Hospital, Nijmegen, The Netherlands

**Keywords:** Multiple sclerosis, Disease-modifying drugs, Patient decision aid, Prototype development, Shared decision making, Alpha testing

## Abstract

**Background:**

Since decision making about treatment with disease-modifying drugs (DMDs) for multiple sclerosis (MS) is preference sensitive, shared decision making between patient and healthcare professional should take place. Patient decision aids could support this shared decision making process by providing information about the disease and the treatment options, to elicit the patient’s preference and to support patients and healthcare professionals in discussing these preferences and matching them with a treatment. Therefore, a prototype of a patient decision aid for MS patients in the Netherlands—based on the principles of multi-criteria decision analysis (MCDA) —was developed, following the recommendations of the International Patient Decision Aid Standards. MCDA was chosen as it might reduce cognitive burden of considering treatment options and matching patient preferences with the treatment options.

**Results:**

After determining the scope to include DMDs labelled for relapsing-remitting MS and clinically isolated syndrome, users’ informational needs were assessed using focus groups (N = 19 patients) and best-worst scaling surveys with patients (N = 185), neurologists and nurses (N = 60) to determine which information about DMDs should be included in the patient decision aid. Next, an online format and computer-based delivery of the patient decision aid was chosen to enable embedding of MCDA. A literature review was conducting to collect evidence on the effectiveness and burden of use of the DMDs. A prototype was developed next, and alpha testing to evaluate its comprehensibility and usability with in total thirteen patients and four healthcare professionals identified several issues regarding content and framing, methods for weighting importance of criteria in the MCDA structure, and the presentation of the conclusions of the patient decision aid ranking the treatment options according to the patient’s preferences. Adaptations were made accordingly, but verification of the rankings provided, validation of the patient decision aid, evaluation of the feasibility of implementation and assessing its value for supporting shared decision making should be addressed in further development of the patient decision aid.

**Conclusion:**

This paper aimed to provide more transparency regarding the developmental process of an MCDA-based patient decision aid for treatment decisions for MS and the challenges faced during this process. Issues identified in the prototype were resolved as much as possible, though some issues remain. Further development is needed to overcome these issues before beta pilot testing with patients and healthcare professionals at the point of clinical decision-making can take place to ultimately enable making conclusions about the value of the MCDA-based patient decision aid for MS patients, healthcare professionals and the quality of care.

**Supplementary Information:**

The online version contains supplementary material available at 10.1186/s12911-021-01479-w.

## Introduction

Multiple sclerosis (MS) is a chronic demyelinating disease of the central nervous system that manifests most often during young adulthood. People diagnosed with the relapsing-remitting disease course of multiple sclerosis (RRMS) experience exacerbations of MS signs and symptoms which can recover over time, though signs and symptoms may remain [1]. If patients have multiple relapses, disability can accumulate, resulting in substantial loss of quality of life [2–4].

RRMS patients and patients diagnosed with clinically isolated syndrome (CIS)—defined by a single event resembling an MS relapse, but in the absence of the MS diagnosis yet [1]—face the decision of starting treatment with disease-modifying drugs (DMDs). DMDs can delay or prevent the accumulation of disabilities by reducing the number of relapses and reducing the number of new (gadolinium enhancing on T1 or new T2) or enlarging lesions on MRI scans of the brain. DMDs can be categorized into first-line DMDs or second-line DMD if treatment with first-line DMDs was not successful [5]. Currently, twelve DMDs are formally authorized for the treatment of RRMS in the Netherlands; three of these have been authorized for CIS [6]. New DMDs are in the pipeline.

A shift from paternalistic decision-making to shared decision-making is taking place in healthcare between the healthcare professional and the patient, which recognizes the importance of both perspectives in the decision-making: medical expertise and experiences with DMD treatment from the healthcare professional’s perspective and personal preferences for treatment options by the patient [7, 8]. The decision for starting DMD treatment is preference-sensitive: it requires a trade-off between treatment benefits and treatment burden in which the patient’s preferences and values should play a key role [8]. Healthcare professionals should invite the patient to participate in the decision-making process, inform patients about their treatment options and elicit the patient’s preferences for the treatment options in order to include these preferences while making a shared decision regarding treatment [9]. Shared decision making could potentially result in better drug use compliance [10]. However, MS patients may have difficulty understanding treatment options. The large number of treatment options and the uncertainties associated with the options in terms of effects complicate the decision-making for healthcare professionals [7]. Moreover, health literacy is an issue for a substantial proportion of the Dutch population [11], and for MS patients the decision to start with a DMD can be even more difficult due to the cognitive and mental symptoms many patients experience [12, 13].

Patient decision aids are not purposed to replace the consultation with the healthcare professional, but to support and enable patients to participate in shared decision-making by explaining the treatment options, their potential benefits and burdens, and help patients to form and communicate their preferences for the treatment options [14]. Patient decision aids have been shown to support patients in feeling more informed, feeling more certain about the decision, and for the decision to be more congruent with the patient’s preferences [14]. Patient decision aids could therefore influence the patient’s preferences and therefore, indirectly, the treatment decision. Accordingly, the adequate development and quality of patient decision aids are important [15, 16].

A couple of patient decision aids are available or are being developed for decisions about DMDs for MS in Canada, Germany, the United Kingdom and the United States, which have different formats (paper-based vs. computer-based) and scopes (starting treatment (yes/no; first-line DMDs only; second-line and first-line DMDs). Patient decision aids for MS developed for one country or region do not necessarily generalize to other contexts, due to potential differences in criteria for patients’ eligibility for DMDs and, possibly, differences in patients’ informational needs. Reports on the effectiveness or developmental process have been published about only few patient decision aids [17–19]. The objective of this paper was to describe our developmental process of a patient decision aid for decisions for all DMDs available for patients with RRMS and CIS in the Netherlands, provide transparency regarding the developmental process and the content of the patient decision aid, and discuss the challenges we encountered in developing such a tool. Transparency in the development and content of the patient decision aid enables the appraisal of whether the developmental process and quality of the patient decision aid are adequate.

## Methods

Recommendations by the International Patient Decision Aid Standards (IPDAS) [15] guided the development of the patient decision aid, which consisted of six stages; these are described below. We applied the principles of a user-centred design, involving the end-users in different stages of the developmental process [20].

### Stage 1: Scope of the patient decision aid

The scope of the patient decision aid was determined within a steering group consisting of three health services researchers and an MS neurologist, verified through consultation of an advisory group consisting of three representatives of patient organisations for MS, three MS neurologists, two MS nurses and an expert in patient decision aid development. The steering group determined *a priori* that the patient decision aid would be based on the principles of multi-criteria decision analysis (MCDA). Key principles for MCDA are that (1) a decision maker has two or more options to fulfil his needs or objectives; (2) alternative options fulfil objectives (or characteristics) to varying degrees and a trade-off between characteristics of the alternative options needs to be made; (3) not one alternative dominates over other alternatives [21]. In MCDA, the decision between alternative options depends on the value put on different characteristics of the options. Using a value measurement model, overall scores or values per alternative option are constructed. These score indicates the extent to which a certain alternative option is preferred compared to the other options.

### Stage 2: Assessment of decisional needs

Focus groups and surveys of the prospective users (MS patients and healthcare professionals) were conducted to assess the patients’ decisional needs, i.e. the information about treatment options that should be provided. Methods have been reported in detail elsewhere [22, 23]. In short, three focus groups were conducted with RRMS patients with prior experience in making a decision about DMDs or using DMDs. Using a nominal group technique, subjects were asked to answer the following question: “What characteristics of DMDs do you feel are important to consider when having to make a decision about DMD treatment?” and to list and define the DMD characteristics that came to mind. Next, subjects individually selected the 10 most important characteristics. All characteristics selected at least once for the top 10 by any of the subjects were then included in a best-worst scaling survey to prioritize the characteristics according to importance in a larger sample of patients and among neurologists and MS nurses. The best-worst scaling survey presented 17 choice tasks. Each choice task consisted of a unique combination of five characteristics derived from the compiled list in the focus groups. The choice tasks were created using a fractional design with Sawtooth SSI Web version 8.20, considering the most efficient design. Further specification regarding the design is reported elsewhere [22]. In each choice task, respondents had to select the most and least important characteristic of DMDs for decision-making. The best-worst scaling resulted in a ranking of DMD characteristics according to their importance in the treatment decision. This ranking guided the inclusion of information in the patient decision aid. The final selection of characteristics and their definitions were presented to the advisory group for feedback, and discussed with two experts in the development of MCDA-based patient decision aids.

### Stage 3: Format

The steering group chose to use MCDA to construct the patient decision aid because of the large number of DMD options and characteristics of DMDs important in the decision. Trading-off multiple characteristics of a number of alternatives could be difficult, especially if patients have cognitive and mental symptoms. The MCDA approach makes the trade-off, which is usually an implicit cognitive process, explicit. By combining the importance of characteristics with how well DMDs perform on these characteristics, MCDA provides a summary of how well the treatment options fit the preferences, i.e. the implicit cognitive process is performed by the MCDA tool. Therefore, we hypothesize that the MCDA tool may relieve overall cognitive burden for patients, which could be helpful for patients who might experience cognitive and mental problems. Without any formal support, such trade-offs could be a cognitively burdensome exercise, even for people without cognitive or mental issues, resulting in suboptimal and non-transparent decisions. By ranking the alternative treatment options according to the patient’s preferences [24], the patient decision aid supports patients and healthcare professionals in directing the focus of the deliberation in terms of characteristics and alternative options to discuss.

### Stage 4: Review and synthesis of evidence

To determine the performance of each DMD on efficacy characteristics, an inventory of all pivotal randomized controlled studies of DMDs for MS was made through a database search. Moreover, the database search identified relevant meta-analyses synthesizing the results of pivotal studies on the efficacy of DMDs in comparison with a placebo or other DMDs. The Cochrane Controlled Register of Trials (CENTRAL) was searched (latest update 12 June 2017) for reviews using the term “multiple sclerosis”. Due to a lack of evidence of the effects of certain DMDs in comparison with a placebo in pivotal studies, network meta-analyses of DMDs were identified through a database search in Medline (Pubmed) (latest update 12 June 2017), a search for health technology assessment reports in databases of the National Institute for Health and Care Excellence and of the Centre for Review and Dissemination and through experts. Network meta-analyses include direct comparisons of DMDs to a placebo and indirect comparisons of DMDs to other DMDs to estimate the DMDs’ performance in comparison with a placebo [25]. The network meta-analysis was selected based on search date, acceptability of quality and comprehensiveness in terms of relevant DMDs, according to the outcome measures of interest (i.e. the effect on relapse rate and disease progression). Effect estimates of DMDs in the selected network meta-analysis [26] were compared with results from other network meta-analysis for verification. Effect estimates for DMDs not included in the meta-analysis were derived from pivotal studies of the DMDs included in its Cochrane review.

Since the network meta-analysis included only estimates for effects on relapse rate and disability progression, patient-reported effects on quality of life (QoL), cognitive capabilities and fatigue were derived from randomized controlled studies identified through a review of QoL [27], irrespective of the measurement instrument used. Outcomes for cognition and fatigue were based on relevant subdomains of the QoL instruments wherever included and reported. If the data of subdomains were not reported, corresponding authors—and in case of no response, first and/or last authors—were contacted to retrieve additional data.

The effect on MRI outcomes was defined—in agreement with the advisory group—as “no gadolinium-enhancing lesions and no new or enlarging T2-hyperintense lesions on the MRI” [1]. Heterogeneity in operationalization, measurement and reporting of MRI outcomes hinders the synthesis of data for many DMDs, and was therefore not included in the selected network meta-analysis. Other network-meta-analyses identified through the initial search were screened for relevancy of MRI outcomes. If DMDs were not included or only partial information was available in the network meta-analyses, missing data were obtained from the Cochrane reviews or the pivotal studies. If no pooling of data from multiple studies was conducted, the project group pooled the data using RevMan version 5.

Ease of use, safety profiles and common side effects were based on the Summary of Product Characteristics of each DMD and on data from the Dutch Healthcare Institute. Information for contra-indications because of comorbidity or other use of medication were identified using summary of product characteristics and information provided by the Dutch Healthcare Institute (www.farmacotherapeutischkompas.nl).

### Stage 5: Development of prototype

A prototype of the patient decision aid was then developed. In MCDA decision aids, performances of each treatment on the characteristics as derived from literature need to be translated into a performance score between 0 and 1 [24]. A performance score of 1 represents optimal performance on this characteristic, while a score of 0 represents no effect or no evidence available. For efficacy on relapses, disease progression, MRI and time to MS diagnosis, linear functions based on relative risk and hazard ratio were assumed. For example, a change in relative risk on relapses of 0.65 to 0.80 would result in an equal change in performance score as would a change in relative risk of 0.25 to 0.40. For efficacy characteristics based on patient-reported outcomes, linear functions were defined based on effect size (Cohen’s d). If no data were available from network meta-analysis or the pivotal studies, or if no significant difference between the DMD and placebo was found, the performance scores of efficacy characteristics was set to zero. Performance scores for common side effects were estimated by calculating the DMDs’ weighted risk for side effects. For DMDs’ performance on safety profiles, a rule was defined according to the categorization of first line and second line medication, which was approved by two clinicians from the advisory group: scores of 0.9 were set for first-generation first-line DMDs, scores of 0.8 were set for second-generation first-line DMDs—since experience in practice and thus information about long-term consequences is more limited in comparison with first-generation first-line DMDs—and scores of 0.6 for second-line DMDs, with the exception for natalizumab scoring 0.8 if a patient has been negatively tested for the JC virus (and therefore having low risk on progressive multifocal leukoencephalopathy). The performance scores for ease of use were not pre-determined, since an objective assessment of the difficulty of using each DMD could not be made for all patients. Therefore, a patient-centred approach was applied for determining these scores. Each patient who uses the patient decision aid rates his/her own performance scores for ease of use per DMD by rating the expected burden of using the DMD on a scale of 0 (not difficult at all) to 10 (very difficult). Performance scores for all characteristics, with the exception of ease of use, were verified by two MS neurologist (EW, WV).

### Stage 6: Alpha pilot testing

Iterative alpha pilot testing to evaluate the comprehensibility and usability of the patient decision aid was conducted—in accordance with the IPDAs recommendations—with healthcare professionals (n=3 (MB, EW, WV)), patient representatives (n = 2 (MK, JS)) and an expert in patient decision aid development (TW) from the advisory and project group. Additional patients were recruited using convenience sampling via advertisements on the social media platform of a patient organization, *MS Vereniging Limburg*. Patients with prior experience with making a decision about DMDs or using DMDs (either with RRMS, CIS or secondary progressive MS (SPMS) and interested in participating in the study were asked to contact the researcher (IK). Patients who were thinking of changing their MS treatment were not eligible. Due to low response, snowball sampling was used in addition to the advertisements, via a patient representative in the advisory group and a member of the patient organization. A patient representative asked other eligible patients to participate. Patients were recruited until data saturation was reached.

A researcher (IK) visited the patients at home and healthcare professionals in their work environment. Patients and healthcare professionals were instructed to go through the patient decision aid in the presence of a researcher, and to verbalize any comments, thoughts or difficulties they had regarding the wording, functionality and usability of the information provided. The researcher took field notes of any comments, and noted answers to prompt questions seeking clarifying comments and observations about the functionality of the patient decision aid. A topic list was prepared before the interview to ensure that all relevant topics were addressed. If topics had not been fully addressed after the respondent had gone through the patient decision aid, the researcher asked additional questions. Field notes were analysed for generalizable themes: remarks regarding text, scope of the patient decision aid, content, sources, functionality, usability and layout, and resulted in points for improvement concerning specific aspects of the patient decision aid. Contradicting results were discussed in the project group until consensus about the needed improvements was reached. Revisions were made and the adapted prototype was tested with other patients until no new substantial comments concerning the usability or comprehensibility of the patient decision aid came up.

## Results

### Stage 1: Scope of the patient decision aid

Patients diagnosed with RRMS or CIS were the target users of the patient decision aid. The patient decision aid includes all brand name and generic DMDs available for patients with RRMS and CIS in the Netherlands, i.e. interferon beta-1b, interferon beta-1a IM, interferon beta-1a SC, peginterferon beta-1a, glatiramer acetate, teriflunomide, dimethyl fumarate, natalizumab, alemtuzumab, fingolimod, cladribine and ocrelizumab. The patient decision aid also includes the option of no drug treatment, which is the reference treatment in the patient decision aid. In the absence of an up-to-date national clinical guideline for MS in the Netherlands, the selection of options was informed by the Dutch Healthcare Institute [6] and expert opinions from members of the advisory group.

### Stage 2: Assessment of decisional needs

Nineteen patients with RRMS (79% female) with a mean (standard deviation, SD) age of 46.8 (8.8) years and a disease duration of 9.5 (8.4) years contributed through focus groups to the identification of important characteristics of DMDs with regard to decision-making on treatment. In total, 34 attributes were identified and defined, and 27 attributes were ultimately included in the best-worst scaling survey. The survey was administered to 185 patients (87% female, with a mean (SD) age of 42 (9.6) years and mean (SD) disease duration of 6.4 (5.9) years, and to 27 neurologists and 33 MS nurses. According to these patients and healthcare professionals, the effect on disease progression, QoL and relapse rate were the most important characteristics for consideration in the treatment decision, followed by safety—according to healthcare professionals—or the severity of side effects—according to patients. The importance scores of all characteristics are presented elsewhere [22, 23]. Patients’ and healthcare professionals’ rankings of characteristics were compared [23]. Although there were small differences in place in the ranking, the rankings of the most important characteristics were similar. Therefore, no differentiation in weighting of the perspectives needed to be made. Comparison of the average rankings of the highest ranked characteristics according to patients and according to healthcare professionals did not reveal substantial differences, and, therefore, no differentiation in weighting of the perspectives needed to be made After assessment of the importance of the characteristics, the characteristics were categorized by the project group to match criteria for MCDA, i.e. avoidance of overlapping with other criteria and preference dependence (the importance of one criterion is not dependent on the performance of an alternative on another criterion) [24, 28]. To avoid preferential dependence, characteristics related to common side effects and administration of the DMD were combined into one category each. Because of overlap with “influence on daily life”, the administration-related characteristics were combined in a new characteristic “ease of use”, which included administration method, frequency, duration, the possibility of drinking alcohol and the ability to drive a car. Relapse rate and severity of relapses were combined into “effect on relapses”. To minimize the overlapping of “effect on MS symptoms” with “effect on disability progression”, the former was split into the most common and burdensome symptoms, specifically “effect on fatigue” and “effect on cognition” as assessed from the patients’ perspective. The effect on disability progression was defined as the change in EDSS score, assessed by a healthcare professional. “Effect on QoL” was included separately, as the domains included in QoL measures vary substantially, meaning that not all measures include fatigue and cognition specifically. The characteristics included and omitted from the patient decision aid are presented in Table 1. Ultimately, nine characteristics of DMDs for RRMS patients were included. For CIS patients, a tenth characteristic was included after consultation with the advisory group, i.e. “time to definite MS diagnosis”. The advisory group agreed with the selection of the characteristics.

**Table 1.** DMD characteristics included and excluded in the patient decision aid

Characteristic	BWS RIS Mean (SD)	New categorization of characteristic
*Included in the patient decision aid*		
Effect on relapse rate	7.76 (2.58)	Effect on relapses
Effect on the severity of relapse	7.39 (2.32)	
Safety	6.04 (2.95)	Safety (risk of severely disabling and life-threatening adverse events)
Uncertainty about long-term consequences	4.58 (2.76)	
Required monitoring	0.55 (1.18)	Required monitoring
Severity of side effects	7.63 (2.11)	Risk of common side effects
Type of side effects	5.00 (2.71)	
Duration of side effects	3.74 (1.97)	
Influence on life style	5.31 (2.92)	Ease of use
Mode of administration	1.58 (2.69)	
Frequency of administration	0.68 (1.38)	
Duration of administration	0.20 (0.24)	
Effect on disability progression	9.64 (1.16)	Effect on disability progression (based on EDSS)
Effect on QoL	9.21 (1.45)	Effect on QoL (based on PRO)
Effect on development of plaques in the brain	7.31 (2.52)	Effect on development of plaques in the brain
Effect on current MS symptoms	7.32 (1.97)	Effect on fatigue (based on PRO)
		Effect on cognition (based on PRO)
*Omitted from the patient decision aid*		
Effect on life expectancy	4.81 (3.13)	
Pace of effect	3.18 (2.19)	
Interaction with other medication	1.72 (1.86)	
Insurance coverage	2.71 (2.87)	
Mode of action of DMD	0.99 (1.25)	
Total DMD costs	0.86 (1.27)	
Further development of DMD	0.87 (0.94)	
Composition of DMD	0.18 (0.36)	
Use of DMD among other MS patients	0.34 (0.56)	
Ease of travelling	0.29 (0.87)	
Contact person at pharmaceutical company	0.10 (0.28)	

BWS, best-worst scaling; DMD, disease-modifying drugs; EDSS, Expanded Disability Status Scale; MS, multiple sclerosis; PRO, patient-reported outcome; QoL, quality of life; RIS, relative importance score; SD, standard deviation

### Stage 3: Format

The underlying algorithms needed in MCDA to estimate the weightings of characteristics and produce rankings of alternatives require a computer-based delivery to enable the embedding of MCDA in a patient decision aid. The patient decision aid was, therefore, built in a survey software combined with an MCDA-software, which allowed for the immediate alignment of the patient’s preferences with the ranking of treatment options.

### Stage 4: Review and synthesis of evidence

The search in Medline (Pubmed) identified 28 records, of which 7 network meta-analyses were assessed as potentially relevant for the outcome “effect on relapses” and “effect on disease progression”. Three additional health technology assessment reports including network meta-analyses [29–31] were identified through experts. The supporting information includes a table (Additional file 1: Table S1) presenting the identified studies according to the search date, the DMDs included in the network meta-analysis and the outcomes included. The most comprehensive and recent network meta-analysis was selected [26]. Studies have shown that generic glatiramer acetate is as effective as brand glatiramer acetate and has similar side effects [32]. Therefore, outcomes for the generic DMD were equated to the effects and adverse effects of the brand.

The review for the effects of DMDs on QoL identified 14 RCTs reporting on any QoL measurement instrument for measuring the effects of the DMDs of interest. In these RCTs, nine different QoL-instruments were used. Reporting on the patient-reported outcomes QoL and its subdomains fatigue and cognition was often incomplete and inconsistent. QoL-instruments were used in 35 instances, of which in only 11 instances differences in change scores from baseline to the follow-up point between the DMD and the comparator were (partly) reported on subdomains of the QoL-measures. Contacting authors did not result in the inclusion of any additional data of interest in the patient decision aid, mainly due to low response rates. Since the meta-analysis and QoL-review did not include data for cladribine, parameter values for effect measures for cladribine were based on data aggregated by Giovannoni et al. [33] or from the pivotal study [33, 34].

For MRI outcomes, the network meta-analysis published by the Canadian Agency for Drugs Technology and Health was assessed as most relevant, based on the operationalization of the outcomes in terms of the proportion of patients with gadolinium-enhancing lesions and with new or enlarging T2-hyperintense lesions; this meta-analysis included data for natalizumab, dimethylfumarate, fingolimod, teriflunomide and interferon beta-1a SC. Additionally, a review reporting additional data on MRI outcomes was identified through a database search (Medline (Pubmed)) Through the additional database search specifically for MRI outcomes, a review reporting additional data on MRI outcomes was identified [35]. For five DMDs (alemtuzumab, dimethylfumarate, fingolimod, ocrelizumab and teriflunomide), the data of multiple studies were pooled. For three DMDs (glatiramer acetate 40mg, interferon beta-1a SC and interferon beta-1b), no data or data for only one of the dimensions of MRI outcomes were found.

### Stage 5: Development of prototype

**Fig. 1:**
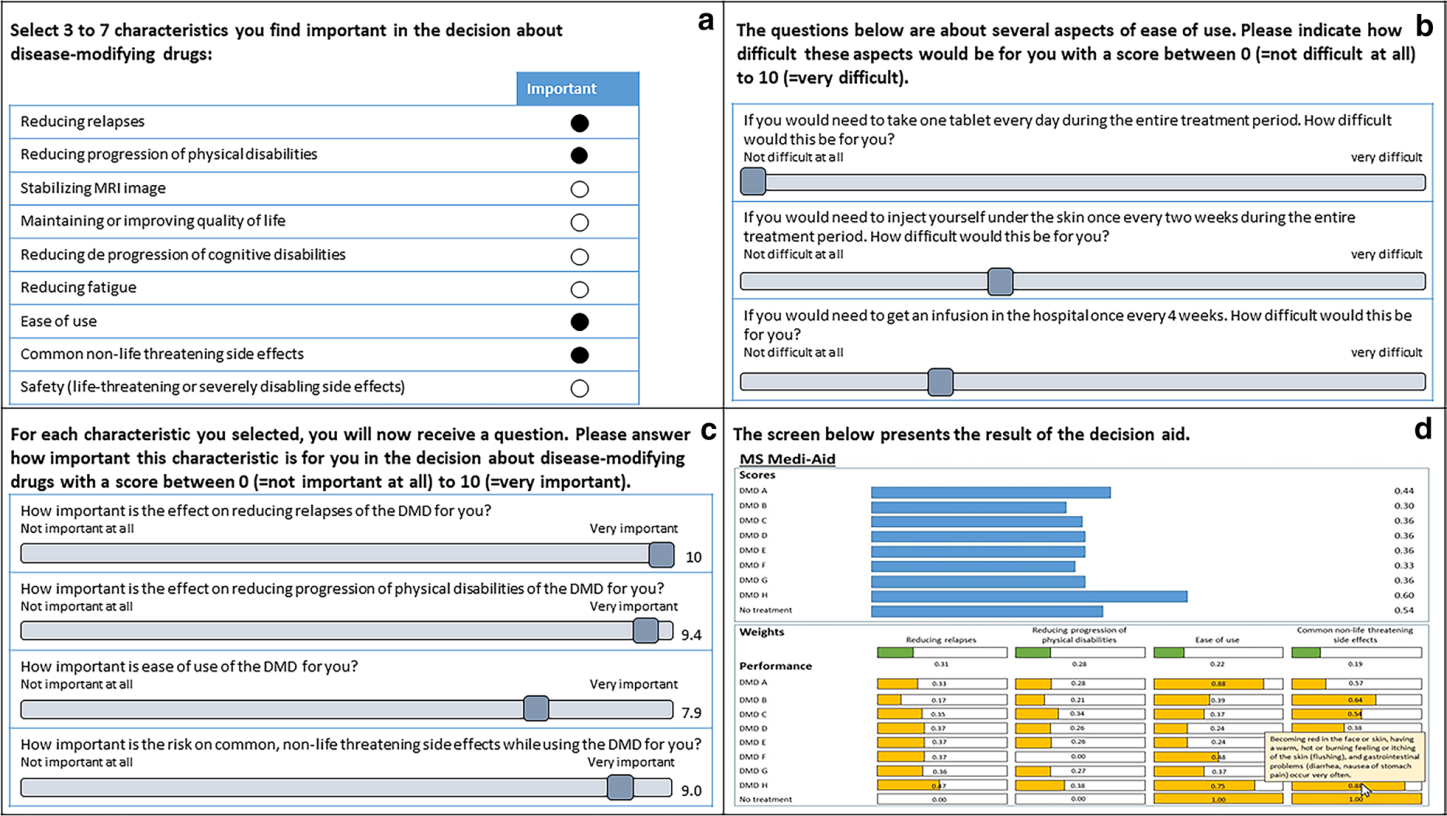
Screen shots of the patient decision aid for MS^*^. ^*^The screenshots have been recreated from the Elicia/Annalisa software for translation of the the Dutch content to English. Therefore, the layout may deviate slightly from the layout in the Elicia/Annalisa software.

The patient decision aid was embedded in the software Elicia/Annalisa (Maldaba) for MCDA-support tools as described in Dowie et al. [36]. Figure 1 shows several screen shots, translated from Dutch to English for the purpose of this publication, illustrating the format and content of the first prototype of the patient decision aid. The patient decision aid consisted of four parts.

*First*, general information is provided about MS, description of the different MS disease courses and the treatment options for RRMS. General information about the treatment options includes a listing of all available options, an explanation of the trade-off in treatment characteristics that patients and their healthcare professionals need to make, and a description of the treatment characteristics.

*Second*, the patient needs to answer a number of questions about their personal demographic characteristics and medical history relevant to the selection of suitable treatment options based on recommendations for clinical practice [6]. These characteristics are gender, type of MS, whether first-line and/or second-line treatment options are considered, comorbidity, current medication use, if the patient is pregnant or breastfeeding or wishes to have children within a year. Type of MS, severity of MS, comorbidity and the use of current medication affected the type of DMDs presented to the patient in the following information.

*Third,* the patient decision aid contains a value elicitation exercise to determine what treatment characteristics are important for the patient to consider in his/her treatment decision and to attach weights to each characteristic. The value elicitation exercise consists of two parts. Initially, the patient selects three to seven characteristics he/she wants to include in his/her decision (Fig. 1a). Next, the patient rates the importance of each characteristic for the decision on a scale from 0 to 10, with 10 being very important, using sliders (Fig. 1b, c). A direct rating method was selected by considering the cognitive burden for patients and the precision required [28].

*Fourth*, a ranking of treatment options is presented according to their suitability to the patient’s preferences. The ranking is presented as separate bars for each DMD. Each bar is compiled by combining the performance scores and the standardized weights of the characteristics according to the individual patient using a weighted-sum model. The weighted-sum model is a simple and intuitive value measurement model [36] often used for MCDA. The overall scores for DMDs are constructed by summing partial scores for characteristics of the option [24]. The partial scores are calculated by weighting the performance of the DMDs on characteristics according to the importance of these characteristics in the decision, i.e. multiplying the importance of each characteristic according to the patient by the performance of the alternative option on the characteristic. The overall score is thus calculated as follows: $overall\; score\; for \;alternative\; option = \sum w_{i} \times p_{i}$, with w representing the standardized value or weight for characteristic i, and p representing the performance of alternative *j* for characteristic *i.*

In the software, the bar representing the overall score for each DMD is broken down in fragments illustrating the contribution each characteristic makes to the overall score of the DMD. The ranking is purposed to stimulate a discussion between the patient and the healthcare professional about the treatment options by showing the patient’s preferences for treatment characteristics, the performance of the treatment options on each treatment characteristic and how these two relate to each other visually in bars (Fig. 1d). Using pop-ups, the performance of treatment options is explained in text.

For each DMD a performance score per characteristic was calculated. To calculate a weighted risk of the common side effects for each DMD, first, an inventory of all the common side effects associated with any of the DMDs and the associated risks was made. Over 300 different side effects were identified. Two members of the project group (IK and PJ) clustered related or similar side effects into broader categories to reduce the extensive number of side effects to 36 categories. The risk of experiencing a common side effect during the use of a DMD, i.e. the proportion of patients experiencing a side effect among those on the treatment, was assigned a score between 0 (=high risk) and 1(=low risk): 0=reported in ≥10% of patients using the DMD; 0.2= 1-10%; 0.4= 0.1-1%; 0.6= <0.1%; 0.8= incidental report; 1=never reported. Users of the patient decision aid were able to indicate which side effects, with a maximum of five, they would prefer to avoid. Next, patients rated how burdensome these side effects would be for them. The performance scores of DMDs were calculated accordingly: the risk score per side effect was multiplied by the rating for burden and the scores across all side effects were added per DMD and divided by the maximum possible score (i.e. score of 36 in case of no side effects at all).

One of the neurologists indicated during the verification process that the relative performance on MRI outcomes of the first-line DMDs were too high or too low in his experience. This could result from the heterogeneity in operationalization, measurement and reporting the MRI outcomes in pivotal studies, resulting in missing or only partial available MRI outcome estimates. Of the first-line DMDs, only for teriflunomide was sufficient data available for both aspects considered in the MRI outcome. Therefore, in absence of more precise data, no distinction in performance on MRI outcomes was made for these DMDs, and the performance scores for first-line DMDs were equated to teriflunomide.

For DMDs’ performance on safety profiles, first line, first generation DMDs scored 0.9 (relatively safe); first line, second generation DMDs scored 0.8 (due to less extensive experience with the DMD); and second line DMDs scored 0.6. Following suggestions of two neurologists, an exception was made for natalizumab, with regard to the JC-virus status of patients. If patients were negative, the performance of natalizumab was set at 0.8, as the associated risk of serious adverse events is substantially lower.

### Stage 6. Alpha pilot testing

Alpha testing was conducted in three rounds. First, a neurologist, an MS nurse and three patients were asked for feedback. Accordingly, revisions were made to the patient decision aid. Next, 10 additional patients were interviewed and observed. The characteristics of patients included in rounds 1 and 2 are presented in Table 2. No new major comments came up in the last three interviews, indicating that data saturation was reached. Last, the neurologist from the first round, two additional neurologists and an expert in patient decision aid development provided feedback. The healthcare professionals had a lot of experience in the treatment of MS patients with DMDs, were from different hospitals in the Netherlands and were currently working in MS care, except for one neurologist, who was working as neurologist-researcher in MS. Respondents indicated that the patient decision aid has the potential to be a valuable addition to the decision-making process. However, interviews and observations of respondents also identified issues and areas for improvement of the patient decision aid; these were clustered in three overall themes: content and framing, weighting methods for the importance of characteristics according to the patient, and the presentation of the result of the patient decision aid (Table 3). These three themes are discussed below.

**Table 2.** Characteristics of patients (n=13) involved in the alpha testing

		N (%)^a^
Gender	Female	10 (77)
	Male	3 (23)
Age (years)	Mean ±SD	53.9 ±9.0
	Range	35-64
Highest educational level	Pre-vocational education	3 (23)
	Vocational education	3 (23)
	Higher education	7 (54)
MS type	RRMS	11 (85)
	SPMS	2 (15)
Time since MS diagnosis (in years)	Mean ±SD	17.0 ±11.6
	Range	1-38
Experience with DMDs	Yes	13 (100)
	No	0 (0)

MS, multiple sclerosis; RRMS, relapsing-remitting multiple sclerosis; SD, standard deviation; SPMS, secondary progressive multiple sclerosis

^a^Unless otherwise specified

**Table 3.** Major comments and adaptations in the alpha test

Round	Major comment	Adaptations made
*Weighting method for importance of DMD characteristics by patient*		
1	Direct rating does not force patient to differentiate in importance of characteristics	Observations of rating by patients in round 2
2	Several patients rated all characteristics as very important	Alternative weighting methods (i.e. Simple Multiattribute Rating Technique (SMART) and Point Allocation) were incorporated in the patient decision aid as a try-out.
3	Other weighting methods were found to be too complex	Direct rating was applied
*Usability of sliders*		
2	Intervals of 1 decimal are too small	Check boxes replaced the sliders
2	Sliders are difficult for scoring zero	
2	Sliders are difficult if patient’s coordination is reduced	
*Patient’s ability to answer questions*		
1	Patients cannot decide on their eligibility for second-line treatment.	Framing of questions adapted: "Are you willing to accept higher risks of severe adverse events, for more efficacy?"
	Patients might not be able to answer questions regarding other medication use.	Explanation of medications was added
2	Too difficult to answer which type of medication to include	Question whether to include all DMDs was deleted
	Questions about comorbidity, other substances/medication, MS type are too difficult for patients	Since a 'don't know' option was available, no adjustments were made
3	Patients need instructions on how to answer questions about MS type and type of DMDs	Patients may receive a note summarizing their medical history or the healthcare professional fills out the question for the patient
*Rating side effects*		
1	Do not include all 36 side effects. It could scare people	List was reduced to the 10 most common side effects
2	The list of side effects puts off: "I don't want to get any of them"	
3	No comments were made	
*Disease courses*		
1	Include the progressive types of MS and add illustrations to clarify the differences in disease courses	Information and figures were added
2	Figures should illustrate the decline in abilities, instead of the increase in disabilities	Contradictory reactions. Some participants found the explanations about the disease courses clear with the figures as they were, some wanted a change. Figures were kept as they were to connect to the verbal explanation of the disease course, which is also in terms of increasing disability. Further evaluation in beta testing
3	Consider whether to illustrate the decline in abilities, instead of the increase in disabilities	
*Summary page*		
1	The healthcare professional needs to know how the patient’s answers affected the selection of DMDs.	Summary page was added, including information about DMD selection based on the patient’s eligibility
2	no additional comments	
3	no additional comments	
*End screen*		
1	The amount of information on the end screen is overwhelming	Information on weighting and performance scores of the DMDs was made optional. Instructions were added to explain the additional functions to patients.
2	The instructions are too elaborate and difficult to understand	The instructions were adjusted a number of times and tested with new patients. Visual instructions were added in figures which showed how to access additional information. Still, instructions were too difficult for all patients to understand the whole end screen. The end screen was divided in two parts. The first part presents only the rankings of the DMDs and explanations focus only on explaining these rankings. Access to the second part, which explains in depth how the rankings were compiled, is optional
3	The patient decision aid may not be suitable for patients to go through individually, except for highly-educated patients. Consider whether an MS nurse should guide the patient in the use of the patient decision aid	Beta test should show whether the guidance of an MS nurse is feasible and desirable for patients
*Additional reading*		
2	Preference for the possibility of reading more information on DMDs of choice	An option to select three DMDs to read more about was included. The patient decision aid provides a schematic overview of the selected DMDs, according to the characteristics
3	No additional comments were made	

#### Content and framing

Three major issues were identified by healthcare professionals and/or patients regarding the content and framing. First, while the patient decision aid initially included only general information on RRMS and CIS, a healthcare professional in round 1 of the pilot test indicated that general information about the progressive disease courses would be useful in painting a complete picture for the patient. This information was added to the patient decision aid, including figures sketching the extent to which disabilities could increase over time in the different disease courses. In round 2, most patients agreed that the information about all disease courses was useful. Some patients commented that the figures would be more logical if they would illustrate how physical abilities could decrease instead of how physical disabilities could increase. No majority for one of the two possibilities was identified during the interviews with the patients, nor with the professionals in round 3. Therefore, figures presenting increase in physical disability were used, as this was regularly used in the clinical practice of the healthcare professionals involved.

A second comment on the patient decision aid made by a nurse in round 1 and reinforced by a number of patients in round 2 concerned the query about selecting the side effects which the patient would like to avoid. The list of 36 side effects was too overwhelming for patients and off-putting. When confronted with the list, a number of patients stated: “I don’t want to get any of them”, wanting to select all side effects. Because many patients had this reaction to the question, the list of 36 side effects was reduced to ten side effects that occur very often or often when using any of the DMDs. The question about side effects was adapted to: “Which side effect would you definitely want to avoid?” The algorithm for calculating the performance score of DMDs on the side effects was adapted. Any DMD with a risk of occurring in 1% or more of patients using the DMD would score zero on the side effects. In round 3, no new comments on the cognitive burden for patients of answering this question were raised by the professionals.

As a third issue, in all rounds, the neurologists, MS nurses and the patients indicated that questions regarding the type of MS, comorbidity, other substance or medication use and whether patients are eligible for first-line and/or second-line medication could be difficult to answer, despite adaptations to the formulation of these questions in between rounds, with the goal of easing the understandability of the questions. Two solutions were considered appropriate and were discussed in rounds 2 and 3 with patients and healthcare professionals: (1) the patient receives a note summarizing their medical history, which the patient can copy into the patient decision aid; or (2) the healthcare professional (treating neurologist or MS nurse) fills out the specific questions on medical history before the patient uses the patient decision aid. Beta pilot testing would need to show which method is most feasible for implementation.

In addition, during all rounds a number of textual remarks were processed to clarify information or to match explanations currently given by healthcare professionals in clinical practice. Examples are to illustrate cognitive disabilities with examples and to elaborate on the role of monitoring in securing safety during second line drug use.

#### Weighting methods for the importance of characteristics according to the patient

During the first round of the alpha testing, a neurologist questioned whether the direct rating method would be the best approach as a weighting method as “all patients would rate the characteristics as very important.” In round 2, observations and interviews with patients did not identify any problems for patients to weight characteristics using the direct rating methods, but showed indeed that the direct rating method resulted in relatively flat distributions in importance scores. The majority of the patients rated the selected characteristics with the same or almost the same score, most often a 9 or 10, representing “very important”. In round 3, several other methods were considered and discussed with the professionals. Other methods, such as Simple Multi-Attribute Rating Technique and Point Allocation [28], were discussed with the professionals as alternatives to direct rating, since these methods specifically incite patients to compare the characteristics according to their relative importance. However, the neurologists, expert in patient decision aid development and the project group regarded these methods as too difficult and cognitively burdensome for patients. The risk of flatter distributions of importance scores for the characteristics in the direct rating method was therefore accepted, as characteristics could in theory be equally important for the patient in the decision.

In round 2, it became apparent that the initial format for patients to weight characteristics using sliders was problematic in several ways: the interval of 1 decimal was distracting; scoring 0 was difficult because respondents had to move the slider from zero and back; and in terms of coordination the sliders were difficult to use, especially using the mouse pad of a laptop. Patients indicated that they would prefer simply selecting the weight between zero and ten using check boxes. Adaptations were made accordingly.

#### Presentation of the result of the patient decision aid

**Fig. 2:**
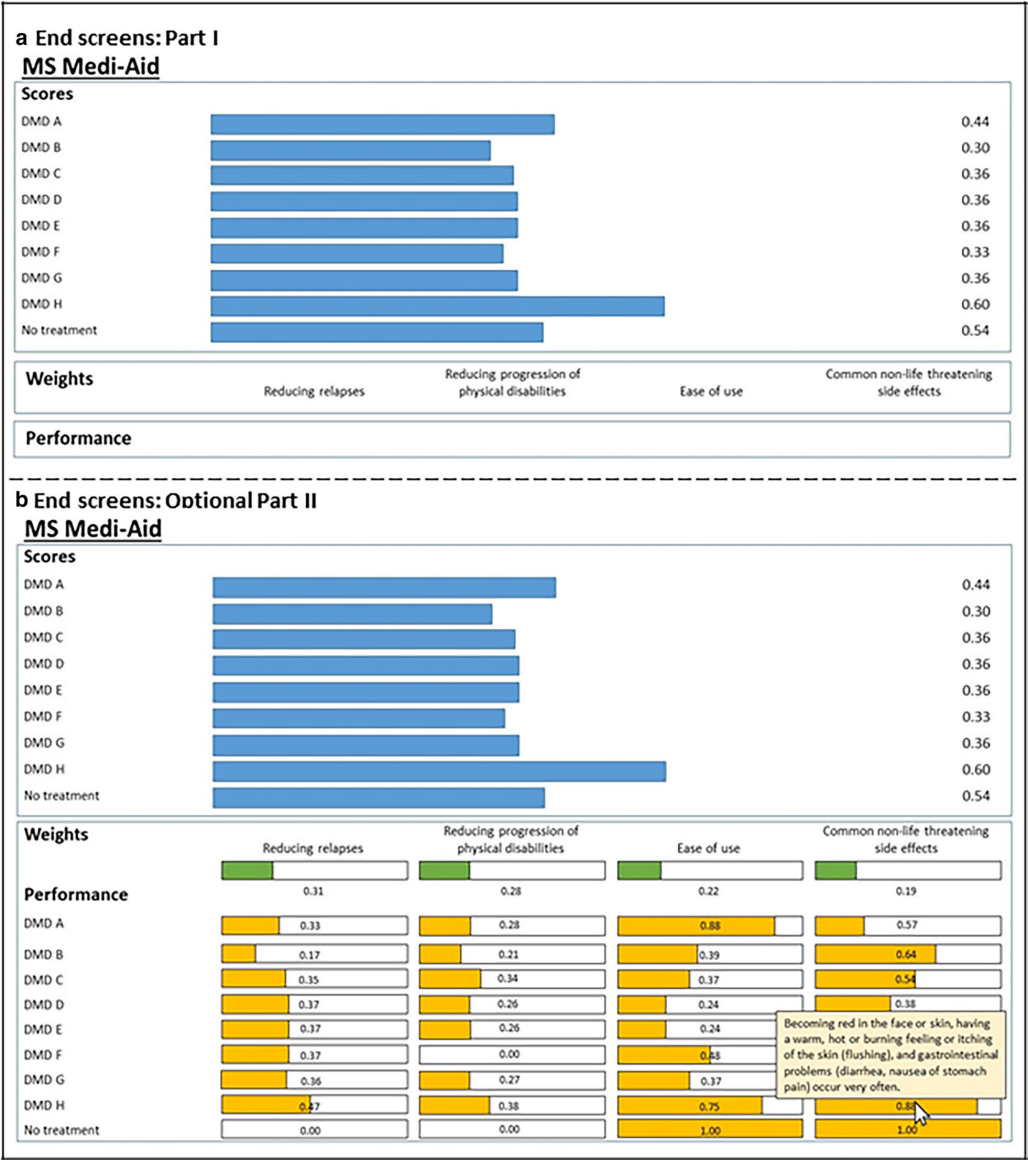
End screens: Parts I and II

Three major challenges were identified regarding the presentation of the result of the patient decision aid: ensuring that the patient understands the ranking of the DMDs according to the preferences, ensuring that healthcare professionals understand how DMDs have been filtered out based on the patient’s eligibility, and ensuring that patients have both sufficient and structured information about the most suitable DMDs. From round 1, it became apparent that the result screen presenting the ranking of DMDs, the weightings of the characteristics and the performance levels of the DMDs according to the characteristics could be overwhelming for patients. Therefore, the result screen and its explanation underwent a number of adaptations throughout the pilot testing between the different rounds and, within round 2, between smaller groups of patients. After round 1, the choice was made to present only the ranking of the DMDs to the patient and to collapse the information about the weighting of the characteristics and the performance scores of the DMDs. The patient is be able to call up the additional information, explaining the ranking of the DMDs based on the weights of the characteristics as rated by the patient and the performance of the DMDs on the characteristics, by clicking a button. As a result, the bars illustrating the weights and performance of DMDs on the characteristics fold out in the same screen (Fig. 2b). Instructions explaining the functionalities of the result screen were given one page earlier. This format was tested in subsequent rounds with patients. The most prominent challenge was to instruct patients adequately about the functionalities while considering the information processing capacities of MS patients: elaborating on the explanation was too tiresome, yet shorter explanations resulted in misunderstandings. In the different iterations, the instructions were constantly adapted in response to the patients’ comments by adding figures, changing the layout to ease reading and adapting formulations of the instructions. Even so, most patients needed additional instructions from the interviewer to fully understand the results. Once the results were fully understood, the patients were enthusiastic about the richness of the patient decision aid: “It helps to deepen my knowledge and prepare me for the consultation.” In round 3, the project group made the choice to present a simplified version of the result screen to the patient first, only presenting the rankings of the DMDs, without the ability to fold out extra information (Fig. 2a). This limited the amount of instructions—presented just above the ranking—that the patient needs to understand all of the functions of the result screen. Next in the patient decision aid, the patient is asked whether he/she want to learn more about how the ranking was compiled. A subsequent screen includes the patient’s weights for characteristics and the DMDs’ performances on the characteristics in bars, with pop-ups explaining the bars (Fig. 2b). This way, the patient decision aid was adapted to fit patient preferences and cognitive abilities in processing the amount of information about the DMDs better. After these adjustments, the patient decision aid expert and the neurologists still questioned whether the patient decision aid would be suitable for use by patients with low or middle levels of education. Beta pilot testing should show whether supervision by a trained MS nurse is needed when going through the patient decision aid and whether this delivery format is feasible in clinical practice.

In round 1, the neurologist commented that an understanding is needed of how questions concerning the patient’s eligibility for the DMDs (e.g. type of MS, comorbidity) affect the selection of the DMDs in the patient decision aid for each individual patient. Therefore, a summary page (Additional file 3: Table S3) was included at the end of the patient decision aid stating all criteria that affected the selection of DMDs suitable for the patient, the patient’s answers and which DMDs were omitted as a result from the patient decision aid. If a patient answered “don’t know”, this was also presented. The summary page was further evaluated in the following rounds, and no additional comments were made by the respondents.

The result screen (the ranking and optionally the weighting of characteristics and the performance scores of the DMDs), the schematic overview of the DMDs’ characteristics and the summary of the assessment of the patient’s eligibility for the DMDs can be printed and/or sent to the healthcare professional.

In round 2, a couple of patients commented that they would prefer additional information on the result screen, summarizing the most suitable DMD options. One patient commented: “I would not be able to choose based on this [the ranking].” Therefore, in deliberation with the patients, a function was added through which the patient could select the three DMDs he/she wants to read more about, based on the initial ranking of the DMDs. This information, which is identical to the information in the pop-ups for the performance bars, is provided in a tabular form presenting the characteristics of the three DMDs side by side.

#### Other minor remarks

A number of minor remarks regarding the format, scope and functionality of the patient decision aid were made in all rounds of the pilot testing, and adaptations were made accordingly where possible (Additional file 2: Table S2). However, technical functionalities of the software limited certain desired adjustments, such as spacing between text, enlarging check options, accentuating the cursor on the screen, using progress bars instead of page numbers, including menu tabs to switch between chapters, using pop-ups for more information and additional reading. Before beta-testing of the patient decision aid, a switch to different software will be made so that additional functionalities and layout requirements can be incorporated.

## Discussion

### Summary

A systematic approach was used to develop an online patient decision aid about DMDs for the treatment of MS. The approach consisted of defining the scope, assessing users’ decisional needs, establishing a format, reviewing the evidence, developing a prototype and iteratively alpha testing the prototype. The alpha test showed that the prototype had some issues regarding the content and framing, the methodologies used for weighting the options and the end screen. Adaptations were made accordingly, but room for improvement remains and some issues should be further studied since only alpha testing has been conducted thus far.

### Strengths and weaknesses of the patient decision aid

The developed patient decision aid differs from most other patient decision aids in the approach used. The MCDA-based approach was applied as we hypothesized that this approach averts putting a high cognitive burden on the MS patient to study and understand all available DMDs for the treatment of MS by performing an explicit trade-off of (conflicting) characteristics according to the patient’s preferences. Using the approach in our patient decision aid, the patient is able to make a selection of treatment options he/she wants to read more about. The deliberation and consultation with the healthcare professional can then be focused on the selected DMDs, as these best fit the patient’s preferences. The application of the MCDA approach did, however, also introduce new difficulties for patients in terms of understanding the ranking of the treatment options and how ranking was compiled. Changes in the delivery of the patient decision aid could support patients in this. Whether the MCDA-approach actually reduces overall cognitive burden should be further studied, preferably by comparing decision-making with the MCDA-based patient decision aid to decision-making without the decision aid or with the decision aids without decision analysis.

The application of the MCDA approach does mean that the patient decision aid may not fulfil all criteria for patient decision aids as established by the IPDAS [37]. For example, in the patient decision aid it is optional for patients to read information about all treatment options, including the probabilities on outcomes, risks and procedures involved; this information is provided per DMD is after the preference elicitation. Because the patient decision aid explicitly includes the patient’s preferences in the ranking, it is only optional for the patient to read the information. This approach increases the personalization of the patient decision aid to the patient’s informational needs, but could decrease the patient’s knowledge gain about *all* treatment options in comparison with other patient decision aids.

The MCDA approach resulted in other challenges as well, specifically concerning the translation of the outcome estimates into performance scores between zero and one. Comparability of the DMDs for a specific characteristic is difficult when clinical data is lacking or has not been completely reported, such as the data for MRI outcomes. Performance scores were in that case set to zero, the same score if no significant difference between the treatment option and placebo was found. We, however, acknowledge that lack of evidence is not the same as no efficacy, and lower levels of evidence, such as results from observational studies and expert opinions. Validation of the performance scores with a larger group of clinical experts is needed to increase the objectivity of the scores. Moreover, inclusion of additional data from Phase IV studies, including observational ones, could enhance the overall picture of all treatment options.

Another limitation of the development of the patient decision aid is that the study sample included solely patients experienced with making a decision about DMD, while the target users also include treatment-naïve patients. The reason for this was to not burden patients recently diagnosed and currently in the decision making process, a vulnerable and demanding time, with an untested prototype for which a certain level of usefulness had not been established. Although the study group has been in the same position as the target users, they were not at the point of participation in the study. The study sample was asked to recall what information and support of a patient decision aid they would have needed in hindsight. However, we acknowledge that the study group may differ in certain characteristics from the target users of the patient decision aid, such as time since MS diagnosis, age, knowledge about MS and treatment options and cognitive capabilities. Consequently, the study sample might not recall the time of decision making accurately or their needs might have changed over time, e.g. less concerned with aspects such as long-term consequences or consequences for child-bearing or fathering, and would therefore not accurately reflect the needs of the target users. The patient decision aid is targeted at treatment-naïve patients considering to start DMDs and treatment-experienced patients considering to switch or re-start a (new) DMD. While the study sample will reflect the latter population more closely, the former population may have different needs. Further evaluation of a more advanced version of the patient decision aid with treatment-naïve patients will, therefore, be needed.

The alpha testing was primarily aimed at establishing the content, usability and comprehensibility of the patient decision aid. Further development of the patient decision aid is still needed, such as improvements in layout and functionalities (which were limited by the software), and the appropriate delivery of the patient decision aid needs to be tested with regard to maximizing understanding of the results (i.e. whether the aid should be used by the individual patient or used under the supervision of a trained nurse).

This patient decision aid contains components specifically for MS patient in the Netherlands. The patient decision aid could, however, be transferred to other countries. Adaptations to the content of the patient decision aid should be made for it to fit country-specific contexts, for instance regarding eligibility criteria for DMDs and, possibly, regarding aspects that should be included (e.g. out-of-pocket expenses might be important in countries where DMDs are not fully covered by health insurance).

### Recommendations and implications for implementation and further research

First, in absence of a clinical guideline from the Dutch Society for Neurology during the period of development, the patient decision aid has been developed based on the best available evidence in the literature and following recommendations from the Dutch Healthcare Institute. An updated guideline was recently published. To facilitate implementation, it is recommended that patient decision aids relate closely to clinical guidelines [38]. Therefore, the patient decision aid should be regularly reviewed and updated as soon as a clinical guideline has been completed or adjusted, and formal relations with these clinical guidelines should be established. Moreover, the field for MS treatments is developing quickly, making the decision for treatment only more complex as new treatments come to market. Keeping the patient decision aid, and for that matter the clinical guidelines, up to date is challenging, but of great importance. The online format of the patient decision aid ensures that changes to its content, i.e. new treatment options, changes in eligibility criteria or new evidence about outcomes and burden, will reach users immediately. However, monitoring these developments will be challenging. Structures should be set up to ensure immediate updates when new evidence becomes available.

Second, the developmental process, including the alpha testing, confirmed that shared decision making about DMDs for RRMS is complex. Moreover, we learned that developing an MCDA-based patient decision aid to support the process has its challenges, and that still some obstacles need to be overcome. One of these obstacles is translating evidence into performance scores for certain characteristics, such as safety and MRI outcomes. Safety does not translate easily to a score between 0 and 1 because the data is textual and, although based on among others the summary of product characteristics, the translation introduces subjectivity. A second obstacle is the inconsistency in reporting of outcome measures on DMDs and the gap in high level evidence about the comparative effectiveness of DMDs on a number of characteristics, such as quality of life, MRI outcomes, and cognition. Lower level evidence, such as observational studies or expert opinions could fill these gaps until higher level evidence becomes available. Delphi studies could be useful to reach consensus among experts regarding the performance scores. A number of other aspects still need further work, such as verification of the rankings made by the decision aid, including the influence of uncertainty about effect estimates, and improving understandability of the patient decision aid through evaluation of the required health literacy, adaptation of the delivery of the patient decision aid and validation of the patient decision aid. In addition, the participants in the alpha test were positive about the potential use of the patient decision aid, but since implementation of the patient decision aid was not yet pilot tested, participants could only speculate about the actual added value for shared decision-making. Our choice for MCDA as an approach for the patient decision aid was motivated by its potential to relieve some burden of understanding all available treatment options, developing preferences and matching the preferences with the treatment options. Whether the current patient decision aid lives up to these expectations, or whether obstacles to overcome would rather increase the burden, (e.g. time burden for healthcare professionals in case all patients need guidance in the use of the patient decision aid and the interpretation of its results) cannot be concluded based on the current study. In further evaluation, a beta pilot test would test the feasibility of implementing the patient decision aid in clinical practice with patients actually making the treatment decision, and enables evaluation of the potential benefits of the patient decision aid on the quality of the decision and decision making process, as other patient decision aids have demonstrated [14]. It has also been argued that patient decision aids positively may affect treatment persistence and treatment adherence [39], and therefore increase health outcomes and decrease the use of healthcare resources [40], although evidence supporting this is still limited [14, 40]. If a beta test would establish the feasibility of the current patient decision aid, a randomized controlled trial comparing the patient decision aid to usual care could provide insights into the (cost-) effectiveness.

Afterwards,

## Conclusion

We systematically developed and alpha tested an MCDA-based online patient decision aid about DMDs for MS. MCDA-based decision support tools could be perceived as a black box if the developmental process and content of the tool have not been made transparent. This paper aimed to provide more insight into the developmental process and challenges faced during this process. Issues identified in the prototype were resolved as much as possible, though some issues remain. Whether adaptations in the delivery of the patient decision aid overcomes these issues, should be further studied. Further development is needed, including beta pilot testing to evaluate the feasibility of implementation in clinical practice, to enable conclusions about the value of the MCDA-based patient decision aid for RRMS patients, healthcare professionals and the quality of care.

## Supplementary Information

**Additional file 1: Table S1.** Network meta-analyses identified for efficacy outcomes.

**Additional file 2: Table S2.** Minor comments and adaptations made in accordance with comments in response to the alpha test.

**Additional file 3: Figure S1.** Summary page of the patient decision aid for MS: example of information provided.

## Data Availability

The datasets used and/or analysed during the current study are available from the corresponding author on reasonable request.

## Abbreviations

DMD: Disease-modifying drugs
MS: Multiple sclerosis
MCDA: Multi-criteria decision analysis
IPDAS: International Patient Decision Aid Standards
RRMS: Relapsing-remitting multiple sclerosis
CIS: Clinically isolated syndrome
QoL: Quality of life
SPMS: Secondary progressive multiple sclerosis
