# Supplementary material for: Patient decision aid based on multi-criteria decision analysis for disease-modifying drugs for multiple sclerosis: prototype development
Source: BMC Med Inform Decis Mak. 2021 Apr 9;21:123. doi: 10.1186/s12911-021-01479-w (PMC8033667; doi:10.1186/s12911-021-01479-w)
Supplement: Supplementary file 1 — Additional file 1: Table S1. Network meta-analyses identified for efficacy outcomes. [file 12911_2021_1479_MOESM1_ESM.docx]

Supplementary material

**Patient decision aid based on multi-criteria decision analysis for disease-modifying drugs for multiple sclerosis: prototype development**

I.E.H. Kremer, P.J. Jongen, S.M.A.A. Evers, E.L.J. Hoogervorst, W.I.M. Verhagen, M. Hiligsmann

Table 1. Network meta-analyses identified for efficacy outcomes

|  | Search date | DMDs | Outcome |
| --- | --- | --- | --- |
| CADTH 2014 [1] | Oct 2013 | ATZ (ARR only), DMF, FIN, GA20, IFN (excl. PEGINF), TF | ARR, patients with sustained disability progression (either confirmed after 3 or 6m) |
| Couto et al. 2016 [2] | 09 Nov 2015 | ATZ,DMF, FIN, GA20, GA40 (ARR only), IFNB, PEGIFN, NTZ, TF | ARR, patients with disability progression |
| Del Santo et al. 2012 [3] | NR | IFN, GA, NTZ, FIN vs. placebo | relapse-free rate |
| Fillippini et al. 2013 [4] | Feb 2012 | IFN, GA, NTZ, MTX, Methotrexate, CYC, AZA, IMM, Corticosteroids | Recurrence of relapses, patients with disability progression |
| Fogarty et al. 2016 [5] | Mar 2016 | ATZ, NTZ, FIN, DMF, PEGIFN, GA,20, GA0, IFNB, TF | ARR, patients with confirmed disability progression |
| Hadjigeorgiou et al. 2013 [6] | 12 Nov 2012 | IFNB, TF, GA, NTZ, FIN, MTX, but not vs. placebo | patients free of relapses, patients without MR progression, patients without disability progression |
| Huisman et al. 2013 [7] | 14 Nov 2014 | FIN vs. DMF in highly active RRMS; FIN vs. NTZ in rapidly evolving severe MS | ARR, difference in EDSS change, patients with confirmed disability progression |
| ICER 2017 [8] | 15 Sep 2016 | ATZ, DMF, FIN, GA20, GA40, IFNB, NTZ, PEGIFN, TF, OCR, DAC, RTX | ARR, patients with disability progression confirmed |
| Tramacere et al. 2015 [9] | 30 Sep 2014 | IFN, GA, NTZ, MTX, FIN, TF, DMF, ATZ, PEGIFN, DAC, OCR, LAQ, AZA, IMM | patients with new relapses, patients with disability worsening |
| Zintzaras et al. 2012 [10] | Jan 2011 | FIN, NTZ, RTX, CLAD, IFNB-1a, TF, FINVs. IFNB-1b 250ug/ placebo | Patients free of relapse, Patients without MRI progression, Patients without disease progression |

References

1. Canadian Agency for Drugs and Technologies in Health. CADTH therapeutic review. Comparative clinical and cost-effectiveness of drug therapies for relapsing-remitting multiple sclerosis. 2013 Oct; (CADTH Therapeutic Review vol.1, no. 2b). Available from: <http://www.cadth.ca/media/pdf/TR0004_RRMS_ScienceReport_e.pdf>.

2. Couto E, Hamidi V, Ringerike T, Odgaard-Jensen J, Harboe I, Klemp M. Medicines used for multiple sclerosis - a health technology assessment. Oslo: Norwegian Institute of Public Health, 2016.

3. Del Santo F, Maratea D, Fadda V, Trippoli S, Messori A. Treatments for relapsing-remitting multiple sclerosis: summarising current information by network meta-analysis. Eur J Clin Pharmacol. 2012;68(4):441-8. Epub 2011/11/08. doi: 10.1007/s00228-011-1141-1.

4. Filippini G, Del Giovane C, Vacchi L, D'Amico R, Di Pietrantonj C, Beecher D, et al. Immunomodulators and immunosuppressants for multiple sclerosis: a network meta-analysis. Cochrane Database Syst Rev. 2013;(6):Cd008933. Epub 2013/06/08. doi: 10.1002/14651858.CD008933.pub2.

5. Fogarty E, Schmitz S, Tubridy N, Walsh C, Barry M. Comparative efficacy of disease-modifying therapies for patients with relapsing remitting multiple sclerosis: systematic review and network meta-analysis. Mult Scler Relat Disord. 2016;9:23-30. Epub 2016/09/21. doi: 10.1016/j.msard.2016.06.001.

6. Hadjigeorgiou GM, Doxani C, Miligkos M, Ziakas P, Bakalos G, Papadimitriou D, et al. A network meta-analysis of randomized controlled trials for comparing the effectiveness and safety profile of treatments with marketing authorization for relapsing multiple sclerosis. J Clin Pharm Ther. 2013;38(6):433-9. Epub 2013/08/21. doi: 10.1111/jcpt.12090.

7. Huisman E, Papadimitropoulou K, Jarrett J, Bending M, Firth Z, Allen F, et al. Systematic literature review and network meta-analysis in highly active relapsing-remitting multiple sclerosis and rapidly evolving severe multiple sclerosis. BMJ open. 2017;7(3):e013430. Epub 2017/03/12. doi: 10.1136/bmjopen-2016-013430.

8. Institute for Clinical and Economic Review. Disease-modifying therapies for relapsing-remitting and primary-progressive multiple sclerosis: effectiveness and value.2017 [14 December 2018]. Available from: https ://icer-revie w.org/wp-conte nt/uploa ds/2016/08/CTAF_MS_Final Repor t_03061 7.pdf.

9. Tramacere I, Del Giovane C, Salanti G, D'Amico R, Filippini G. Immunomodulators and immunosuppressants for relapsing-remitting multiple sclerosis: a network meta-analysis. Cochrane Database Syst Rev. 2015;(9):Cd011381. Epub 2015/09/19. doi: 10.1002/14651858.CD011381.pub2.

10. Zintzaras E, Doxani C, Mprotsis T, Schmid CH, Hadjigeorgiou GM. Network analysis of randomized controlled trials in multiple sclerosis. Clin Ther. 2012;34(4):857-69.e9. Epub 2012/03/27. doi: 10.1016/j.clinthera.2012.02.018.
