# Supplementary material for: Patient decision aid based on multi-criteria decision analysis for disease-modifying drugs for multiple sclerosis: prototype development
Source: BMC Med Inform Decis Mak. 2021 Apr 9;21:123. doi: 10.1186/s12911-021-01479-w (PMC8033667; doi:10.1186/s12911-021-01479-w)
Supplement: Supplementary file 2 — Additional file 2: Table S2. Minor comments and adaptations made in accordance with comments in response to the alpha test. [file 12911_2021_1479_MOESM2_ESM.doc]

Supplementary material

**Patient decision aid based on multi-criteria decision analysis for disease-modifying drugs for multiple sclerosis: prototype development**

I.E.H. Kremer, P.J. Jongen, S.M.A.A. Evers, E.L.J. Hoogervorst, W.I.M. Verhagen, M. Hiligsmann

Table 2. Minor comments and adaptations made in accordance with comments in response to the alpha test

| Round | Minor comments | Adjustments |
| --- | --- | --- |
| 1. *Content and framing* | |  |
| 1 | Illustrate cognitive disabilities with examples | Adjustments made accordingly. |
|  | Consistent use of terminology for "relapses" | Adjustments made accordingly. |
|  | Provide nuances in discussing disease progression. | Adjustments made accordingly. |
|  | Differentiate in the occurrence of different side effects. | Adjustments made accordingly. |
|  | Provide the generic drug names first, followed by the brand name in brackets for reasons of independence. | Brands are better known to patients. Therefore, brand name is followed by generic name. |
|  | Elaborate on the role of monitoring programs to improve safety and reduce risks. | Adjustments made accordingly. |
|  | Information about whether the patient is planning to travel in the short term and needs vaccinations would support the consultation. | Adjustments made accordingly. |
|  | Explain second-line medication | Adjustments made accordingly. |
|  | Patients need more information about the process of injecting medication. | Adjustments made accordingly. |
|  | Information about monitoring during use of second-line medication to feel at ease about safety | Adjustments made accordingly. |
|  | Link to useful/reliable patient information | Adjustments made accordingly. |
| 2 | Wish to have children should include option “don’t know” | Adjustments made accordingly. |
|  | Travel plans should include option “don’t know”. | Adjustments made accordingly. |
|  | Choosing characteristics is difficult. “They are all important.” | - |
|  | Pictures presenting the degree of disability progression for explaining natural course of different MS types is confronting. | - |
|  | Figures explaining MS types should be “wavy”. | - |
| 3 | Difficulty distinguishing between characteristics, also after reading additional information. | - |
| 1. *End screen* | |  |
| 1 | Would be informative to see ranking of medication based on a single characteristic | The performance scores per DMD provide this information. |
|  | In result screen, add the possibility to change weights to see their effect immediately. | - |
|  | In result screen, rank medication from most to least fitting to patient’s preferences. | Software limits the ability to make adjustments accordingly. |
|  | In result screen, distinguish between first-line and second-line medication. | Software limits the ability to make adjustments accordingly. |
|  | Explain the direction of the side effects in the result screen as this could be counterintuitive | Adjustments made accordingly. |
| 2 | Repeat characteristics above the performance bars in result screen | Software limits the ability to make adjustments accordingly. |
|  | Remove numbers in performance bars or put them in the bars, not underneath them | Software limits the ability to make adjustments accordingly |
| 3 | Selection of DMD to read more about should be presented under the result screen. The link between the ranking and the selection should also be clearer. | Software limits the ability to make adjustments accordingly. |
|  | Should be able to select DMD in Result screen for further information. | Software limits the ability to make adjustments accordingly. |
|  | End screen seems like a black box. | - |
| 1. *Other* | |  |
| 1 | Provide clinicians with an instructional card about the patient decision aid for use in clinical practice. | Beta-testing |
|  | A separate patient decision aid for CIS might be obsolete because of the earlier diagnosis of RRMS according to the new McDonald diagnostic criteria. | - |
|  | Consider adding treatment options for PPMS and SPMS. | - |
|  | It’s difficult for colour-blind people to distinguish red and green. | Software limits the ability to make adjustments accordingly. |
|  | Online is difficult because I would like to highlight text and go back to it if I need a break. | Option added to print text. Patient decision aid already included option for saving progress. |
|  | Consider whether text can be read out to people. | Beta-testing |
| 2 | Include the option to send results to the specialist. | Beta-testing |
|  | Include the option to print all results or send it to e-mail. | Beta-testing |
|  | Notice of missing answers is unclear. | Software limits the ability to make adjustments accordingly. |
|  | Difficult to select answer options: check boxes are too small | Software limits the ability to make adjustments accordingly. |
|  | It’s difficult to see the cursor on a white screen. | Software limits the ability to make adjustments accordingly. |
|  | Difficulty with returning to patient decision aid after accessing an external link for further reading. | Include information as pop-up in same screen. Software limits the ability to make adjustments accordingly. |
|  | Reactions to questions should appear immediately under the question after answering instead of on the next page. | Adjustments made accordingly. |
|  | Space for summaries or notes would be useful. | Adjustments made accordingly. |
|  | Effect on QoL should be excluded because QoL will never be the same again. | - |
|  | Page numbering at the top of the page is off-putting: “29 pages!” | Software limits the ability to make adjustments accordingly. |
|  | PPMS doesn’t have to be explained. | - |
| 3 | Formulate sentences addressing the reader directly. | Adjustments made accordingly. |
|  | Simplify language. Too much jargon. | Adjustments made accordingly. Before beta-testing the reading level of the patient decision aid will be reviewed. |
|  | Include menu tab that shows new chapters and ability to go back to a previous chapter. | Software limits the ability to make adjustments accordingly. |
|  | Use a progression bar instead of page numbers. | Software limits the ability to make adjustments accordingly |
|  | Switch order of safety and side effects in the presentation of DMD characteristics | Adjustments made accordingly. |
|  | Too much emphasis on ease of use due to additional questions for rating the performance. | Questions concerning rating burden of use positioned after questions concerning weighting of criteria. |
|  | Delete question about alcohol. Puts too much emphasis on it. Include a statement that all DMDs could be used with normal alcohol use. | Adjustments made accordingly. |
