# Supplementary material for: Patient decision aid based on multi-criteria decision analysis for disease-modifying drugs for multiple sclerosis: prototype development
Source: BMC Med Inform Decis Mak. 2021 Apr 9;21:123. doi: 10.1186/s12911-021-01479-w (PMC8033667; doi:10.1186/s12911-021-01479-w)
Supplement: Supplementary file 3 — Additional file 3: Figure S1. Summary page of the patient decision aid for MS: example of information provided. [file 12911_2021_1479_MOESM3_ESM.docx]

Supplementary material

**Patient decision aid based on multi-criteria decision analysis for disease-modifying drugs for multiple sclerosis: prototype development**

I.E.H. Kremer, P.J. Jongen, S.M.A.A. Evers, E.L.J. Hoogervorst, W.I.M. Verhagen, M. Hiligsmann

Figure: Summary page of the patient decision aid for MS: example of information provided

| **Summary**  Based on your answers to the questions, the patient decision aid excluded the medication options for which you are not eligible. You can see your answers below. It is also explained how your answers affected which medication options have been included in the patient decision aid. This summary will help you and your healthcare professional to understand your options during the consultation.  **Type of MS**  Relapsing-remitting MS |
| --- |
| **Type of medicines included**  First-line medication options only *(fingolimod, alemtuzumab, cladribine, and natalizumab have been excluded)* |
| **Other conditions or diseases you are diagnosed with**  Epilepsy *(interferon beta-1b has been excluded)* |
| **Use of medication or other substances**  None |
| **Pregnancy-related aspects**  None |
| **You answered the following questions with “I don’t know”**  Other diagnosed conditions and diseases: progressive multifocal leukoencephalopathy |
| **Side effect you absolutely want to avoid**  Hair loss  Depression and anxiety |

* Translated from Dutch to English for the purpose of this publication
